# Supplementary material for: Metabolic characterization of tumor-immune interactions by multiplexed immunofluorescence reveals spatial mechanisms of immunotherapy response in non-small cell lung carcinoma (NSCLC)
Source: Nat Commun. 2026 Feb 3;17:837. doi: 10.1038/s41467-026-68633-8 (PMC12868679; doi:10.1038/s41467-026-68633-8)
Supplement: Supplementary file 1 — Supplementary Information [file 41467_2026_68633_MOESM1_ESM.pdf]

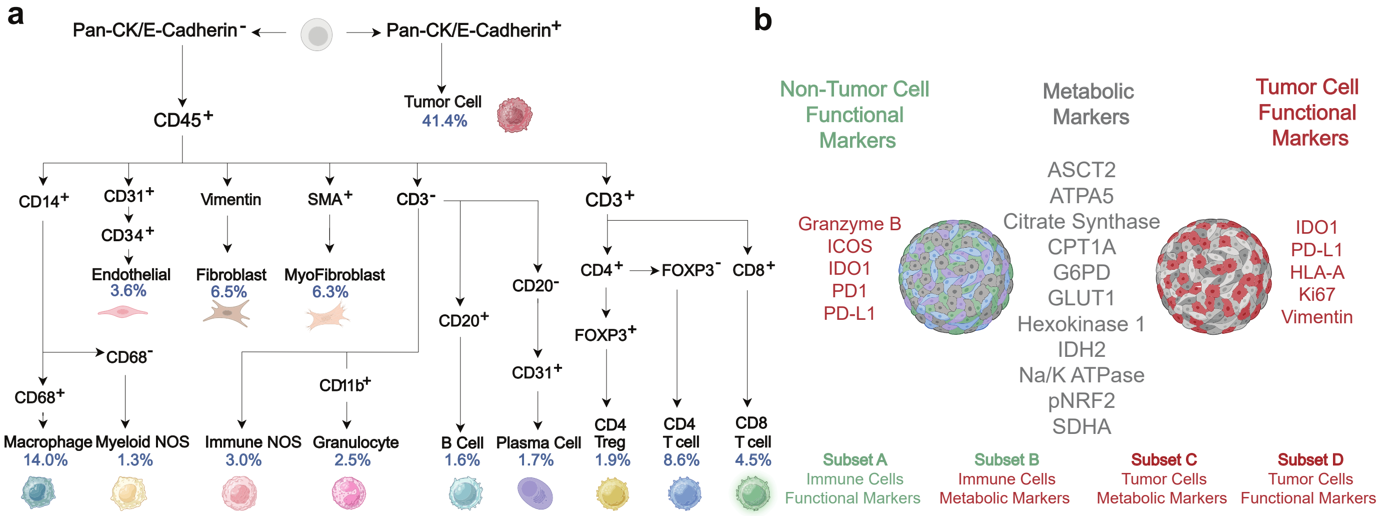

Supplementary Figure 1. Cell typing. a) Flow diagram from cell type allocation based on marker positivity. b) Immune and tumor cell, functional and metabolic markers. Created in BioRender. Kulasinghe, A. (2026) https://BioRender.com/2td43pz.

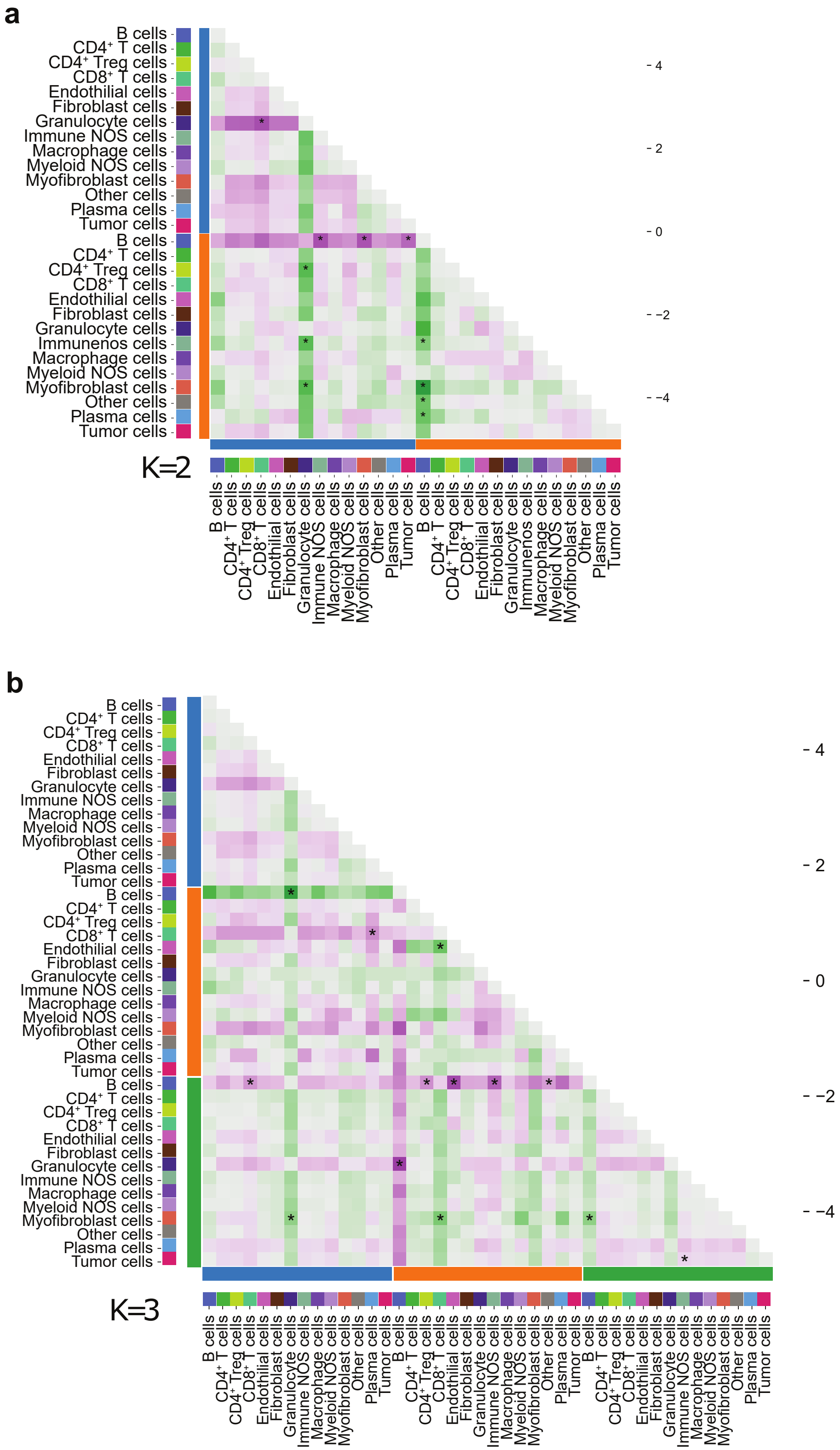

Supplementary Figure 2: Ratios a-b) Cellular ratios within by K=2 (tumor/ stroma CNs) or K=3 (tumor/stroma/interface CNs). Y axis is numerator, x axis denominator

a

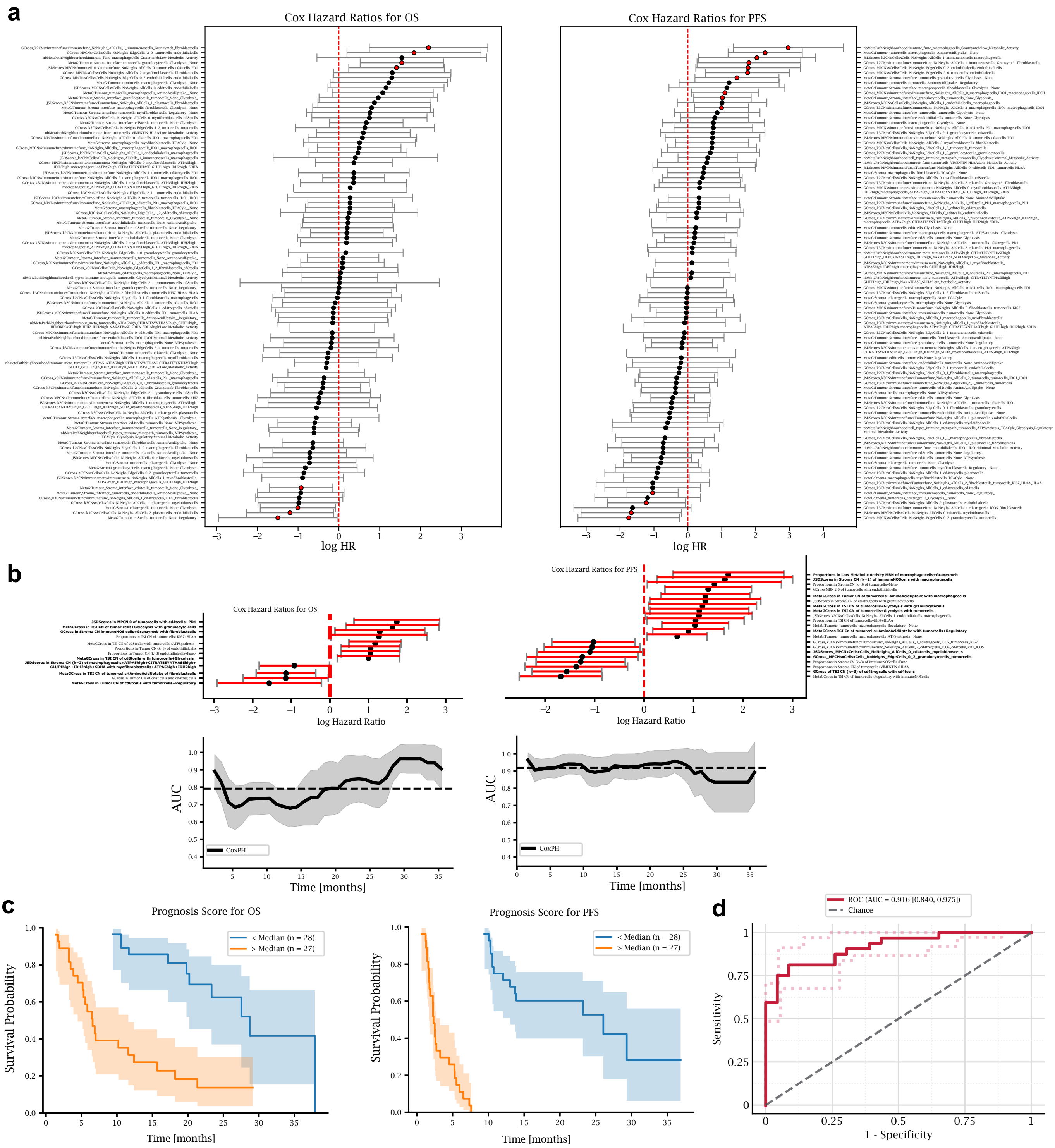

e

	CB6 Selection	PFS Selection	PFS Selection (Balanced Cohort Selection)
**Number of Selected Features**	87	88	100
**Number of Significant Features**	13	22	32
**Overlap with CB6-Selected Features**	N/A	8	37
**Overlap with Significant CB6-Selected Features**	N/A	8	8
**Mean AUC (36 months)**	0.78	0.915	0.914
**YTMA471 Mean AUC (36 months)**	0.73	0.87	0.87
**YTMA404 Mean AUC (12 months)**	0.654	0.935	0.794
**Features Common To All Selection Methods**	**Feature Directionalit**	**KM P-Value**	
GCross in Minimal MBN of macrophagecells IDO1+ with macrophagecells IDO1+	Negative	0.001	
GCross in k2StromaCN of immunenoscells Granzymeb+ with fibroblastcells	Negative	0.001	
GCross in k3InterfaceCN of cd4tregcells ICOS+ with fibroblastcells	Positive	0.02	
Metabolic GCross in k2StromaCN of cd4tregcells with tumorcells+Glycolysis	Positive	0.035	
Metabolic GCross in k2StromaCN of tumorcells+Glycolysis with cd4tregcells	Positive	0.044	
Metabolic GCross in k2TumourCN of tumorcells+AminoAcidUptake with macrophagecells	Negative	0.003	
Proportions in Low_Metabolic_Activity MBN of macrophagecells Granzymeb+	Negative	5.10E-05	

Supplementary Figure 3: Full set of selected features. a) Significant features shown in figure 7 are highlighted with red dots. b) Hazard ratios of significant features that were selected to model PFS using CoxPH fits during Stabl feature selection. Hazard ratios for OS are shown on the left and for PFS on the right. Features also selected to model CB6 are highlighted in bold. Time-dependent AUCs are shown for OS on the left and PFS on the right from k-fold fits. c) Prognosis scores formed by multiplication of feature hazard ratios with normalized feature values predict OS (left) and PFS (right). d) ROC curves using these features to model CB6 using logistic regression show excellent CB6 prediction power in k-fold fits. e) Summary table of features.

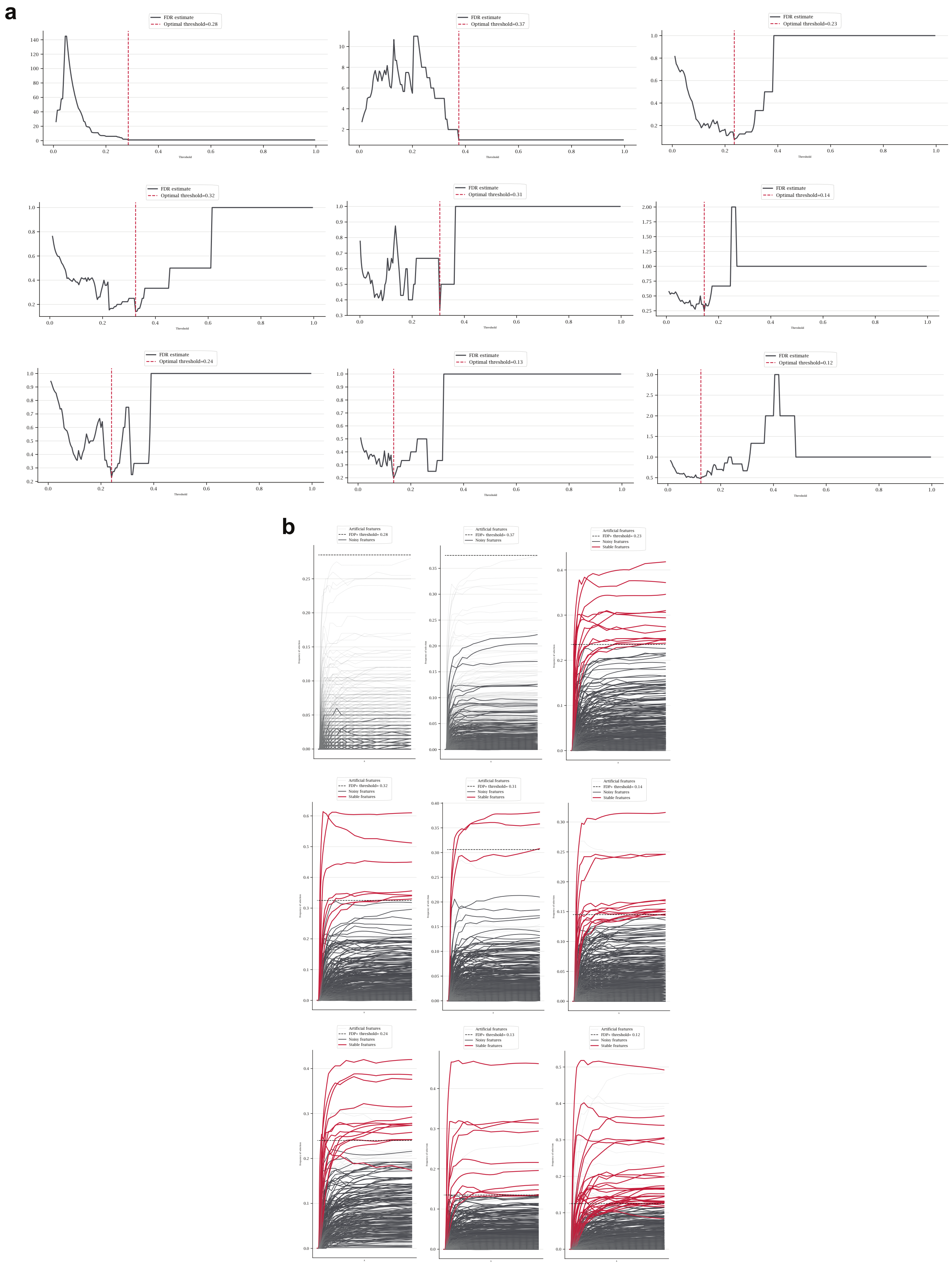

Supplementary Figure 4: False discovery estimates and feature selection paths for each feature family. a) Estimated feature false-discovery rates for each feature family, from left-to-right and top-to-bottom: spatial interaction metrics. b) Feature selection paths across regularization weights for each feature family.

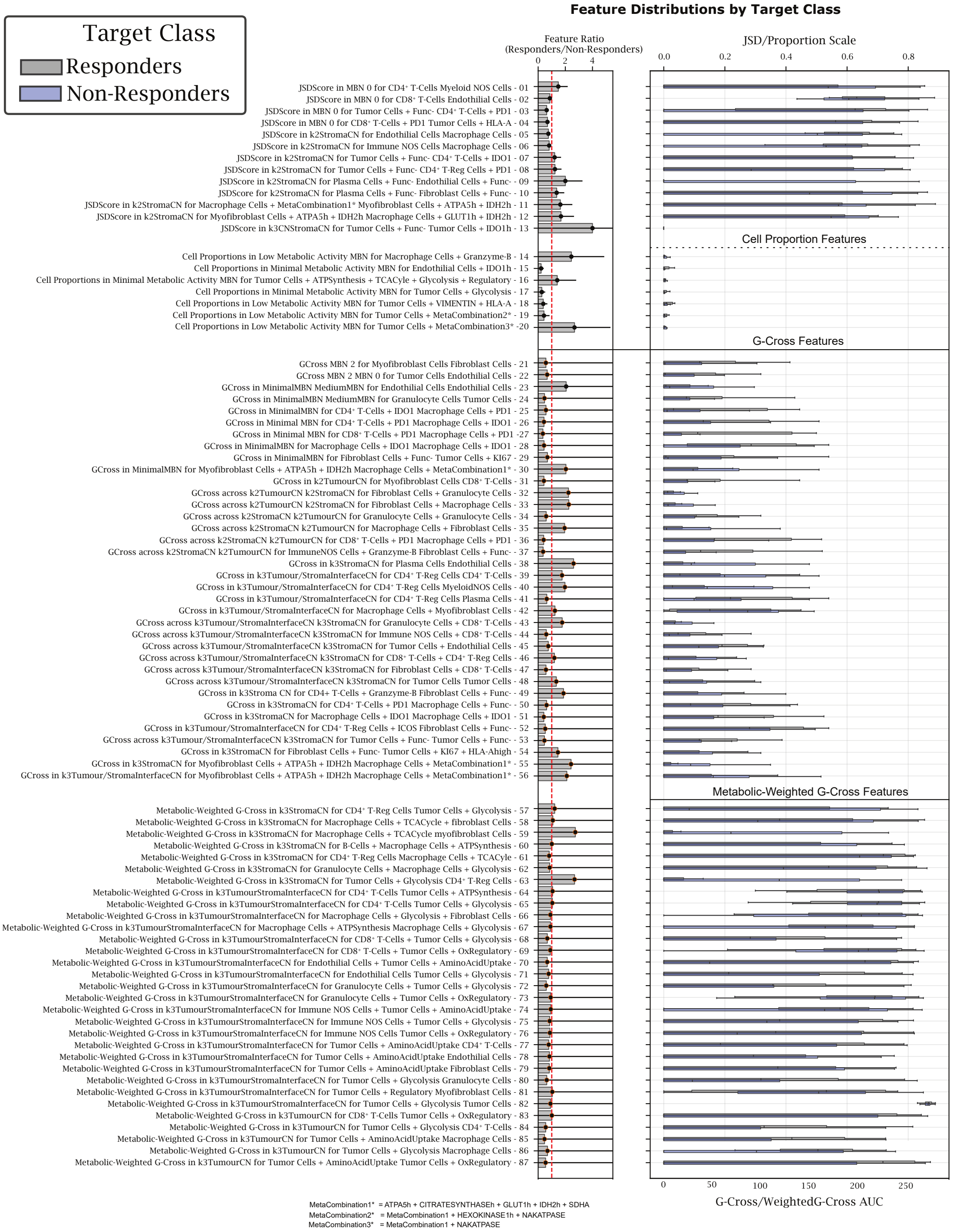

Supplementary Figure 5: Feature distribution by Target Class. Features are separated by feature family; relative importance is shown.

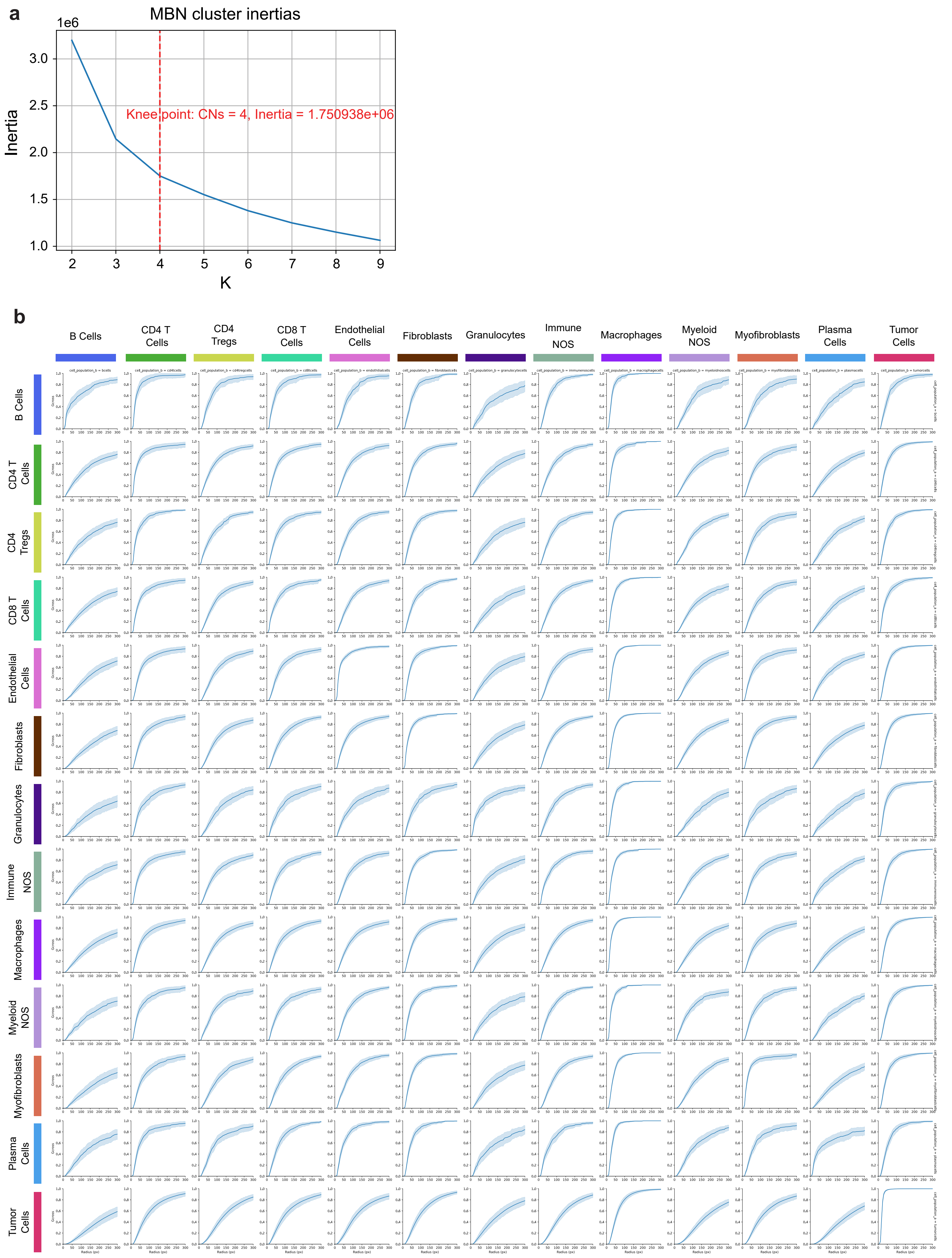

Supplementary Figure 6 a) Optimal number of clusters determined by cluster inertia. b) Globally observed G-Cross curves for cell type combinations sampled from 0 to 300 (150um) pixels in increments of six pixels. Rows represent the query cell type, and columns represent the target cell type. Error bars indicate uncertainty bounds which represent ranges from all cores.
