# Supplementary material for: Metabolic characterization of tumor-immune interactions by multiplexed immunofluorescence reveals spatial mechanisms of immunotherapy response in non-small cell lung carcinoma (NSCLC)
Source: Nat Commun. 2026 Feb 3;17:837. doi: 10.1038/s41467-026-68633-8 (PMC12868679; doi:10.1038/s41467-026-68633-8)
Supplement: Supplementary file 2 — Description of Additional Supplementary Files [file 41467_2026_68633_MOESM2_ESM.pdf]

Description of Additional Supplementary Files

Supplementary Data 1: Deep Learning cell type rules

Supplementary Data 2: Clinical summary table

Supplementary Data 3: Akoya antibodies used in PCF panel
