# Supplementary material for: Metabolic characterization of tumor-immune interactions by multiplexed immunofluorescence reveals spatial mechanisms of immunotherapy response in non-small cell lung carcinoma (NSCLC)
Source: Nat Commun. 2026 Feb 3;17:837. doi: 10.1038/s41467-026-68633-8 (PMC12868679; doi:10.1038/s41467-026-68633-8)
Supplement: Supplementary file 4 — Reporting Summary [file 41467_2026_68633_MOESM4_ESM.pdf]

nature portfolio

Corresponding author(s): Arutha Kulasinghe

Last updated by author(s): 2025/12/09

Reporting Summary

Nature Portfolio wishes to improve the reproducibility of the work that we publish. This form provides structure for consistency and transparency in reporting. For further information on Nature Portfolio policies, see our Editorial Policies and the Editorial Policy Checklist.

Statistics

For all statistical analyses, confirm that the following items are present in the figure legend, table legend, main text, or Methods section.

n/a	Confirmed	
☐	☒	The exact sample size (*n*) for each experimental group/condition, given as a discrete number and unit of measurement
☐	☒	A statement on whether measurements were taken from distinct samples or whether the same sample was measured repeatedly
☐	☒	The statistical test(s) used AND whether they are one- or two-sided<br>*Only common tests should be described solely by name; describe more complex techniques in the Methods section.*
☐	☒	A description of all covariates tested
☐	☒	A description of any assumptions or corrections, such as tests of normality and adjustment for multiple comparisons
☐	☒	A full description of the statistical parameters including central tendency (e.g. means) or other basic estimates (e.g. regression coefficient) AND variation (e.g. standard deviation) or associated estimates of uncertainty (e.g. confidence intervals)
☐	☒	For null hypothesis testing, the test statistic (e.g. *F*, *t*, *r*) with confidence intervals, effect sizes, degrees of freedom and *P* value noted<br>*Give P values as exact values whenever suitable.*
☒	☐	For Bayesian analysis, information on the choice of priors and Markov chain Monte Carlo settings
☒	☐	For hierarchical and complex designs, identification of the appropriate level for tests and full reporting of outcomes
☐	☒	Estimates of effect sizes (e.g. Cohen's *d*, Pearson's *r*), indicating how they were calculated

Our web collection on statistics for biologists contains articles on many of the points above.

Software and code

Policy information about availability of computer code

Data collection	Computer code was not used for data collection.
Data analysis	Open-Source Software<br>1) QuPath (v0.4.2),python (v3.10)<br>Custom scripts were developed for analysis of phenotyped data and are available as shown in data availability

For manuscripts utilizing custom algorithms or software that are central to the research but not yet described in published literature, software must be made available to editors and reviewers. We strongly encourage code deposition in a community repository (e.g. GitHub). See the Nature Portfolio guidelines for submitting code & software for further information.

Data

Policy information about availability of data

All manuscripts must include a data availability statement. This statement should provide the following information, where applicable:

- Accession codes, unique identifiers, or web links for publicly available datasets
- A description of any restrictions on data availability
- For clinical datasets or third party data, please ensure that the statement adheres to our policy

Data availability
Raw image data and formatted anndata used in this study is available at https://doi.org/10.48610/73b218c . Figure source data are provided with this paper.

Code availability
The code used to undertake the analysis is publicly available and has been deposited in the GitHub repository https://github.com/clinicalomx/metabolic-microenvironment-predictors-of-nsclc-immunotherapy-response

Research involving human participants, their data, or biological material

Policy information about studies with human participants or human data. See also policy information about sex, gender (identity/presentation), and sexual orientation and race, ethnicity and racism.

Reporting on sex and gender	The study did not specifically report sex-based differences due to primary objective of developing and validating spatial prognostic signatures with immunotherapy outcomes. Data was stratified by clinical outcomes (progression) and so the influence of sex or gender was not assessed in the study objectives.<br>Justification<br>The absence of sex- and gender-based analyses in this study is due to the study design, which aimed to develop generalisable predictive models in our limited patient cohort, and to maximise the statistical power available. Larger studies in future may consider sex- and gender-specific analysis components if suitable for biomarker discovery goals being investigated.
Reporting on race, ethnicity, or other socially relevant groupings	This information is not available and not required for analysis in our manuscript.
Population characteristics	55 patient samples from advance stage NSCLC treated with PD1 blockade, with median age 67.1. Clinical endpoints included information on overall survival (OS), progression free survival (PFS), as defined by time from start of ICI treatment, and best overall response (BOR) according to RECIST 1.1; complete response (CR), partial response (PR), stable disease (SD) and progressive disease (PD). Progression free survival above 6 months or objective response (partial or complete) was used to define clinical benefit from ICI (CB6).
Recruitment	Tissue samples were retrospectively collected from Yale Cancer Center (YCC). Two tissue microarrays (TMAs) were constructed from independent cohorts from NSCLC patient tumors presenting clinically between 2011 to 2017 (YTMA404) and 2017-2019 (YTMA471).
Ethics oversight	The tissue samples were collected and utilized under the approval of the Yale Human Investigation Committee (HIC), protocol #9505008219, with assurances filed with and approved by the U.S. Department of Health and Human Services.

Note that full information on the approval of the study protocol must also be provided in the manuscript.

Field-specific reporting

Please select the one below that is the best fit for your research. If you are not sure, read the appropriate sections before making your selection.

☒ Life sciences ☐ Behavioural & social sciences ☐ Ecological, evolutionary & environmental sciences

For a reference copy of the document with all sections, see nature.com/documents/nr-reporting-summary-flat.pdf

Life sciences study design

All studies must disclose on these points even when the disclosure is negative.

Sample size	No statistical method was used to predetermine sample size. Sample sizes were based on the availability of tissue samples collected during the specified periods: 2012–2019 for the Yale cohort
Data exclusions	Cohort metadata prior to sample refinement consisted of eighty-two patients with available clinical annotations shown (Fig. 1b). RECIST best overall response (BOR) information was available for seventy-seven patients, consisting of twenty-nine with progressive disease (PD), twenty-six with stable disease (SD), seventeen with partial response (PR), and five with complete response (CR). Samples from non-primary lung tissues were removed (n=10 lymph node, n=2 bowel, n=4 brain, n=1 spinal, n=1 skin), and only specimens taken pre-ICI treatment were considered for analysis (Fig. 1d). The final cohorts consisted of fifty-five patients
Replication	Single patient biopsies (n=55) were analysed. Independent prognostic models were generated and displayed several features common to both independent TMAs
Randomization	In this study, participants were not prospectively randomized into experimental groups. Instead, during model development, samples from the Yale cohort were randomly partitioned into training and testing sets as part of a cross-validation strategy within the LASSO framework.
Blinding	blinding was not used in this study as primary endpoints were considered to stratify patients for analysis

Reporting for specific materials, systems and methods

We require information from authors about some types of materials, experimental systems and methods used in many studies. Here, indicate whether each material, system or method listed is relevant to your study. If you are not sure if a list item applies to your research, read the appropriate section before selecting a response.

Materials & experimental systems

n/a	Involved in the study
☐	☒ Antibodies
☒	☐ Eukaryotic cell lines
☒	☐ Palaeontology and archaeology
☒	☐ Animals and other organisms
☐	☒ Clinical data
☒	☐ Dual use research of concern
☒	☐ Plants

Methods

n/a	Involved in the study
☒	☐ ChIP-seq
☒	☐ Flow cytometry
☒	☐ MRI-based neuroimaging

Antibodies

Antibodies used

Antibody Barcode # Cat No Clone ID Reporter Dye Dilution
ASCT2 BX331 0 0 750 1/200
ATPA5 x 0 0 0 1/200
CD11b BX524 NA NA 647 1/200
CD14 BX037 4450047 AKYP0079 550 1/200
CD20 BX007 4450018 AKYP0049 750 1/200
CD21 BX032 4450027 AKYP0061 550 1/200
CD31 BX001 4450017 AKYP0047 750 1/200
CD34 BX025 4250057 AKYP0088 550 1/200
CD3e BX045 4550119 AKYP0062 647 1/200
CD4 BX003 4550112 AKYP0048 647 1/200
CD44 BX005 4450041 AKYP0073 550 1/200
CD45 BX021 4250099 AKYP0074 550 1/200
CD45RO BX017 4550127 AKYP0059 647 1/200
CD68 BX015 4550113 AKYP0050 647 1/200
CD8 BX026 4250012 AKYP0028 550 1/200
Citrate Synthase BX076 0 0 750 1/200
Collagen IV BX042 4550122 AKYP0083 647 1/200
CPT1A BX500 0 0 647 1/200
E-cadherin BX014 4250021 AKYP0057 550 1/200
FOXP3 BX031 4550071 AKYP0102 647 1/200
G6PD BX468 0 0 750 1/200
GLUT1 B025 0 0 750 1/200
Granzyme B BX041 4250055 AKYP0086 550 1/200
HLA-A BX004 4450046 AKYP0078 750 1/200
ICOS BX054 4550117 AKYP0090 647 1/200
IDO1 BX027 4550123 AKYP0084 647 1/200
Ki67 BX047 4450096 AKYP0052 750 1/200
LAG3 BX055 4550058 AKYP0089 647 1/200
Pan-Cytokeratin BX019 4450020 AKYP0053 750 1/200
PD-1 BX046 4550038 AKYP0070 647 1/200
PD-L1 BX043 4550072 AKYP0103 647 1/200
pNRFR2 BX002 0 0 550 1/200
SMA BX013 4450049 AKYP0081 750 1/200
Vimentin BX022 4450050 AKYP0082 750 1/200

Validation

All antibodies used are Akoya developed. application note for antibody validation for akoya can be found https://www.akoyabio.com/wp-content/uploads/2022/01/Phenocycler_Technical-Note_Validation-of

Clinical data

Policy information about clinical studies
All manuscripts should comply with the ICMJE guidelines for publication of clinical research and a completed CONSORT checklist must be included with all submissions.

Clinical trial registration

This study is retrospective and does not involve a clinical trial

Study protocol

NA

Data collection	. Yale Cancer Center (YCC), New Haven, CT, USA<br>Setting: A comprehensive cancer center within a large academic medical institution.<br>Time Period: Tissue samples were collected retrospectively between 2012 and 2019.
Outcomes	Either objective response (partial or complete) or progression free survival above 6 months was used as the primary endpoint to group patients who received clinical benefit beyond 6 months post treatment (CB6=yes, n=46) vs those who progressed within 6 months

Plants

Seed stocks	NA
Novel plant genotypes	NA
Authentication	NA
